# Metagenomic Analyses of Alcohol Induced Pathogenic Alterations in the Intestinal Microbiome and the Effect of *Lactobacillus rhamnosus GG* Treatment

**DOI:** 10.1371/journal.pone.0053028

**Published:** 2013-01-09

**Authors:** Lara Bull-Otterson, Wenke Feng, Irina Kirpich, Yuhua Wang, Xiang Qin, Yanlong Liu, Leila Gobejishvili, Swati Joshi-Barve, Tulin Ayvaz, Joseph Petrosino, Maiying Kong, David Barker, Craig McClain, Shirish Barve

**Affiliations:** 1 Department of Molecular Virology and Microbiology, Baylor College of Medicine, Houston, Texas, United States of America; 2 Alkek Center for Metagenomics and Microbiome Research, Baylor College of Medicine, Houston, Texas, United States of America; 3 Department of Medicine, Alcohol Research Center, University of Louisville School of Medicine, Louisville, Kentucky, United States of America; 4 Human Genome Sequencing Center, Baylor College of Medicine, Houston, Texas, United States of America; 5 Department of Bioinformatics and Biostatistics, University of Louisville School of Medicine, Louisville, Kentucky, United States of America; 6 Department of Pharmacology and Toxicology, University of Louisville School of Medicine, Louisville, Kentucky, United States of America; 7 Robley Rex VAMC, Louisville, Kentucky, United States of America; Charité, Campus Benjamin Franklin, Germany

## Abstract

Enteric dysbiosis plays an essential role in the pathogenesis of alcoholic liver disease (ALD). Detailed characterization of the alterations in the gut microbiome is needed for understanding their pathogenic role in ALD and developing effective therapeutic approaches using probiotic supplementation. Mice were fed liquid Lieber-DeCarli diet without or with alcohol (5% v/v) for 6 weeks. A subset of mice were administered the probiotic Lactobacillus rhamnosus GG (LGG) from 6 to 8 weeks. Indicators of intestinal permeability, hepatic steatosis, inflammation and injury were evaluated. Metagenomic analysis of the gut microbiome was performed by analyzing the fecal DNA by amplification of the V3–V5 regions of the 16S rRNA gene and large-scale parallel pyrosequencing on the 454 FLX Titanium platform. Chronic ethanol feeding caused a decline in the abundance of both *Bacteriodetes* and *Firmicutes* phyla, with a proportional increase in the gram negative *Proteobacteria* and gram positive *Actinobacteria* phyla; the bacterial genera that showed the biggest expansion were the gram negative alkaline tolerant *Alcaligenes* and gram positive *Corynebacterium*. Commensurate with the qualitative and quantitative alterations in the microbiome, ethanol caused an increase in plasma endotoxin, fecal pH, hepatic inflammation and injury. Notably, the ethanol-induced pathogenic changes in the microbiome and the liver were prevented by LGG supplementation. Overall, significant alterations in the gut microbiome over time occur in response to chronic alcohol exposure and correspond to increases in intestinal barrier dysfunction and development of ALD. Moreover, the altered bacterial communities of the gut may serve as significant therapeutic target for the prevention/treatment of chronic alcohol intake induced intestinal barrier dysfunction and liver disease.

## Introduction

Chronic alcohol consumption is associated with multiple negative health outcomes including alcoholic liver diseases (ALD). Clinical and experimental data have demonstrated that gut-derived endotoxin and endotoxemia play a major role in the development of ALD [1]–[6]. Chronic alcohol consumption mediated endotoxemia can occur due to alterations in the gut microbiota (dysbiosis) and increased endotoxin production, as well as compromised gut barrier function leading to increased intestinal permeability and translocation of bacteria and bacterial products [4], [7]–[9]. Animal experiments have demonstrated that attenuation of ethanol-induced intestinal permeability, endotoxemia and consequent liver injury can be achieved using antibiotics [1] or dietary supplement (oats) [4], [7] or probiotics [5], [7], [10]. This indicates that therapeutic strategies targeting the gut microbiome may be effective in the treatment of ALD. Hence, it is pertinent to characterize the composition of the commensal microbiome and identify the alterations that occur in response to ethanol consumption in ALD. Further, to examine the therapeutic basis of a particular probiotic, it is important to evaluate its beneficial effects on the ethanol-induced alterations in the bacterial composition leading to the attenuation of ALD.

In the present study, we used a mouse model of ALD to examine the temporal effects of chronic ethanol feeding on the commensal bacterial microbiome by identifying the changes in the bacterial communities and their influences on the local environment in the gut. Additionally, we evaluated the role of intestinal microbial community in ethanol-induced ALD by treating the animals with a probiotic strain of lactobacillus – *L.rhamnosus* GG (LGG), and characterizing its effect on the ethanol-induced changes in bacterial communities, intestinal permeability, endotoxemia and hepatic steatosis inflammation and injury. Identification of the changes in the diversity in the bacterial communities in response to chronic ethanol feeding and treatment with LGG was accomplished by deep 16S rRNA gene sequencing using DNA isolated from murine fecal samples. Our results show that chronic alcohol feeding leads to significant shifts in the bacterial community with a marked increase in the gram negative *Proteobacteria* bacteria [11]. Treatment with LGG prevented these shifts and markedly attenuated intestinal permeability, serum endotoxemia and liver injury.

## Methods

### Animal Model and Sample Collection

8–10-week old male mice (C57BL/6N, Harlan, Indianapolis, IN) were pair-fed the Lieber-DeCarli liquid diet containing alcohol (AF, n = 8) or isocaloric maltose dextrin (PF, n = 8). In the first week, all AF groups were given Lieber-DeCarli liquid diet without alcohol. Starting at week 2, alcohol was gradually introduced and increased to 5% (v/v) at the end of the second week and continued until 8 weeks. The Lieber-DeCarli diet contained 17% of energy as protein, 40% as fat, 7% as carbohydrate, and 35% as either alcohol (5% v/v) or isocaloric maltose dextrin (Research Diets, New Brunswick, NJ). *L. rhamnosus* GG (LGG) culture suspension was administered to an additional AF group (AF+LGG, n = 4). : For the probiotic administration, LGG was adjusted in the liquid growth broth at a bacterial density of 1×10^9^ c.f.u/ml. 1 ml of LGG bacterial suspension (1×10^9^ c.f.u) was provided per mouse, per day by mixing it in the liquid Lieber-DeCarli diet. LGG administration was carried out for the last 2 weeks which was initiated at week 6 of 5% (v/v) ethanol feeding and continued until week 8. Stool samples were collected at T1 (Day 0 - baseline, initiation of 5% (v/v) ethanol diet), T2 (end of 6 weeks of ethanol feeding) and T3 (end of study at 8 weeks) for metagenomic analysis. Stool samples were collected weekly throughout the study period and were analyzed for pH. The mice were anesthetized with Avertin and sacrificed at the experimental end point. Blood, liver and intestine tissue samples were collected for histological and biochemical analysis.

#### Ethics statement

This study was carried out in strict accordance with the recommendations in the Guide for the Care and Use of Laboratory Animals of the National Institutes of Health. The protocol was approved by the Institutional Animal Care and Use Committee of the University of Louisville (IACUC - 09018). All surgery was performed under Avertin anesthesia, and all efforts were made to minimize suffering.

### 16S rRNA Gene Sequencing on the 454 FLX-titanium Platform

Bacterial genomic DNA was extracted from fecal samples belonging to mice from each exposure group using the MO BIO Powersoil DNA Extraction Kit (Carlsbad, CA,). In each sample, 750 µl Molecular grade DNAse RNAse free water was added and vortexed for 5 minutes or until the sample was homogenized. Samples were then centrifuged at 2000×g at 4°C for 5 min to remove particulates and the supernatant was aliquoted. The V3–V5 16S rRNA gene variable regions were amplified by PCR using 454 adapter-linked and bar-coded primers 357F and 926R, as described previously [12]. Negative controls (water) and positive controls (*Staphylococcus aureus* USA300 genomic DNA) were included in all steps to control for contamination. Amplicons were purified using a SPRI bead clean-up step, quantitated by picogreen assay, normalized, pooled, and then sequenced on a 454 instrument using the FLX Titanium chemistry.

The sequencing run resulted in approximately 6600 reads per sample with an average read length of 424 bp. The samples were deconvoluted and filtered (retaining those longer than 200 bp with an average quality score of greater than or equal to Q20) to leave 155,979 of the 235,722 reads that passed the initial QC filtering. Chimeric reads were removed using ChimeraSlayer [13]. There were 71,275 reads retained after quality filtering and removal of chimeric sequences with an average read length of 265 nucleotides.

### 16S rRNA Gene Sequencing Analysis

Filtered data was analyzed using both Operational Taxonomic Unit (OTU) and taxonomic binning of classified sequences. The Ribosomal Database Project (RDP) [14] classifier was used to identify the taxonomic classification of the sequence reads. To control for coverage differences between the samples or other conditional sequencing events and to ensure even sampling of the reads, we normalized the number of sequences when comparing samples, so that approximately 4000 reads were randomly picked per sample. The sequences were classified from phylum down to the genus level, starting with 97% confidence cutoff for phylum, a 90% confidence cutoff for family and an 80% confidence cutoff for genus. Mothur [15] and Qiime [16] software packages were used to produce an operational taxonomic units (OTU) table, a distance-based classification and phylogeny from the filtered sequences in order to determine the alpha diversity (the number and abundance of each taxa within a sample) and the beta diversity (the number and abundance of each taxa between samples). Principal coordinate analysis (PCoA) was used to compare microbial community structure using weighted and unweighted UniFrac [17]. Differentially abundant OTUs between populations were compared using Metastats methodology [18]. Metastats uses a permuted two sample T-test to compare the abundance across treatment groups, or Fisher’s exact test for sparsely-sampled data, and employs a FDR test correction to control for multiple testing.

### Liver Histology

Formalin-fixed paraffin embedded liver sections were processed for staining with hematoxylin and eosin and then studied by light microscopy [19].

### Biochemical Analysis

Blood samples from control and alcohol- and probiotic-treated mice were drawn from the dorsal vena cava. Plasma was obtained by centrifuging the blood at 3,000 rpm for 30 min at 4°C. Plasma LPS levels were measured with Limulus Amebocyte Lysate test kit (Lonza, Walkersville, MD) according to the manufacturer’s instructions. Plasma alanine aminotransferase (ALT) was measured using an ALT Infinity Enzymatic Assay Kit (Thermo Scientific, Waltham, MA).

### Fecal pH

Fecal samples (approximately 0.5–1 g) were homogenized with three weight volumes of ice-cold deionized water. Particulate insoluble material were removed by centrifugation (3000×g, 15 min, 20°C), and the pH was measured by a pH electrode [20].

### Real Time RT-PCR Assay

Liver TNFα and intestinal tight junction protein mRNA levels were assessed by real-time RT-PCR. In brief, total RNA was isolated with Trizol according to manufacturer’s protocol (Invitrogen) and reverse-transcribed using the GenAmp RNA PCR kit (Applied Biosystems, Foster City, CA). The sequences of forward and reverse primers are listed in Table 1. The relative quantities of target transcripts were calculated from duplicate samples after normalization to 18s rRNA. using the ΔΔCt method.

**Table 1:** Primer sequences for the targeted mouse genes (RT PCR assay).

Primer set	Forward	Reverse
18s	ctcaacacgggaaacctcac	cgctccaccaactaagaacg
ZO-1	tgggaacagcacacagtgac	gctggccctccttttaacac
Claudin-1	cgggcagatacagtgcaaag	acttcatgccaatggtggac
Occludin	acccgaagaaagatggatcg	catagtcagatgggggtgga
Symplekin	tgagggctgagaaggctgta	cagcacctctgccttgaatc
130	ctggatgctgctgcaactgt	gggcatggagggtcttaatc
Fodrin	cgcatctttttcctcagcag	ccaggacttgctgtcgtctc

### Statistics

Data are expressed as mean ± Standard Error of the Mean (SEM). Statistical significant differences were determined by one-way analysis of variance (ANOVA) followed by Tukey’s post hoc tests. The post hoc t-tests were carried out to examine which two groups were significantly different only if the overall F-test in ANOVA indicated that there was a significant difference among the three groups. Additionally, effect of alcohol feeding on fecal pH over time was assessed, the two-way ANOVA was applied. *P*<0.05 was considered statistically significant. Statistical analysis was performed using GraphPad Prism version 5.01 for Windows (GraphPad Software, Inc, La Jolla, CA).

## Results

### Alcohol Exposure Decreases Bacterial Diversity Over Time

**Figure 1:**
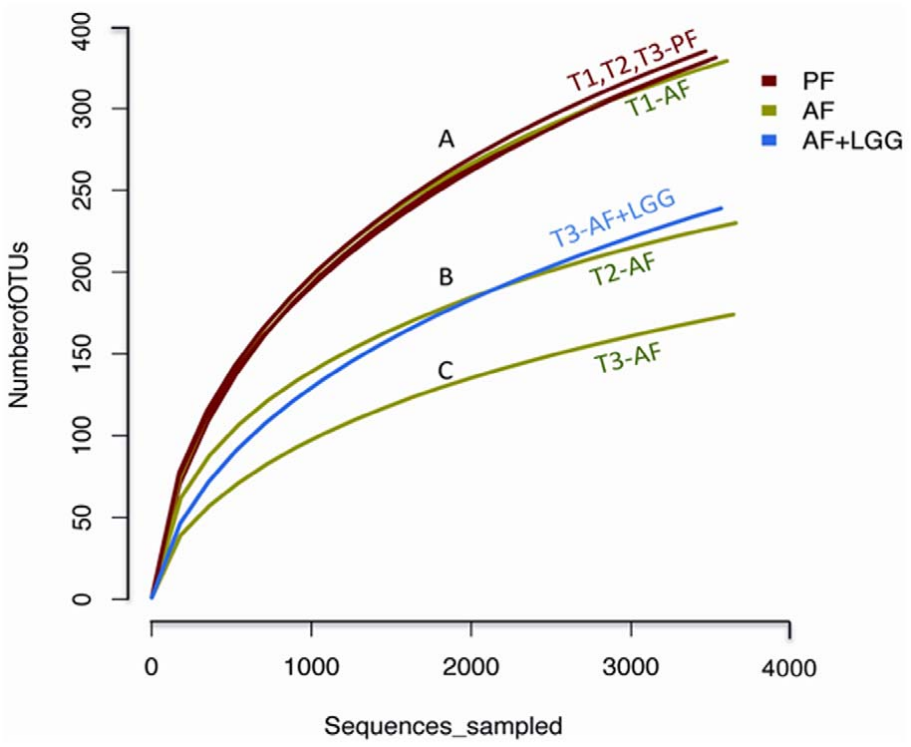
Rarefaction curves indicating the observed number of operational taxonomic units (OTUs) within a sample. Rarefaction curves of OTUs clustered and saturated at different level across exposure groups and indicate the intra-sample diversity. While the baseline PF and AF OTUs cluster together with the PF T2 (end of week 6) and PF T3 (end of week 8) groups (A), the AF T2 and LGG groups (B) saturate earlier than the baseline group show they are less diverse than the baseline group. The AF T3 exposure group (C) saturates the earliest, indicating it is the least diverse sample.

We measured the alpha diversity to examine the number of OTUs, which gives a basic measure of the bacterial diversity within each sample. All the PF samples over time (T1–T3) and AF group at baseline (T1), showed similar rarefaction curves (Fig. 1), indicating that these samples had the most bacterial diversity and the diversity was similar among samples. In comparison, ethanol feeding led to the decrease in the bacterial alpha diversity over time (AF: T2 and T3). Importantly, the rarefaction curves show that LGG supplementation may have limited this decrease in the bacterial diversity richness (AF+LGG: T3).

### Alcohol Exposure Shifts Phylum Representation Over Time

**Figure 2:**
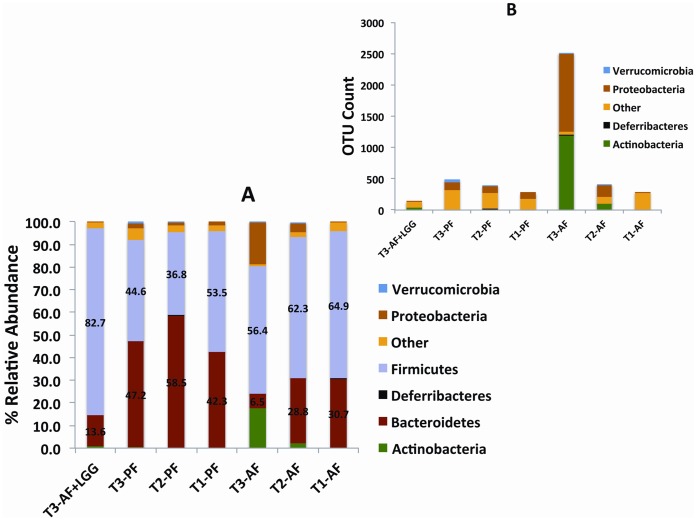
The relative abundance of bacteria phylum by exposure group over time. Time is indicted by time one -T1 (baseline), time two = T2 (end of week 6), time three - T3 (end of week 8). The phylum abundance is indicated by the color bars. The exact percentages of *Bacteroidetes’* and *Firmicutes’* relative abundance are shown in 2A and given by the numbers on the bars. The relative abundance of less common bacterial taxa found after removal of *Bacteroidetes* and *Firmicutes* are shown in 2B. The microbiomes of mice exposed to alcohol at T2 and T3 are characterized by greater abundance of *Proteobacteria* and *Actinobacteria*.

In all PF groups (T1– T3) and baseline AF group (T1), the prominent phyla were the *Bacteroidetes* and *Firmicutes,* where *Firmicutes* was either the dominant phylum or the ratio between phyla was approximately equal (50/50) (Fig. 2). Also, in these groups, the *Proteobacteria* were present at approximately 1–2% and *Actinobacteria* were <1% of the total bacteria identified.

In comparison to PF animals, at the end of 8 weeks (T3), the AF group showed the greatest phyla shifts (Fig. 2A) where there was a reduction in both *Bacteroidetes* and *Firmicutes* and a remarkable expansion of the *Proteobacteria* (from <1% to 19%) and *Actinobacteria* (from 0% to 18%) (Fig. 2B). Importantly, LGG treatment was able to prevent the ethanol-induced expansion of the *Actinobacteria* and the *Proteobacteria* phyla. However, there was a substantial expansion of the *Firmicutes* in the LGG group, which was the most dominant phylum (83% of the total bacteria). Also of note, the ethanol-induced expansion of the phylum *Proteobacteria*, which consists of gram negative bacteria, was decreased in the LGG treatment and the decrease correlated with plasma endotoxin levels (see results below).

### Alcohol Exposure Shifts Bacterial Genus Diversity Over Time

**Figure 3:**
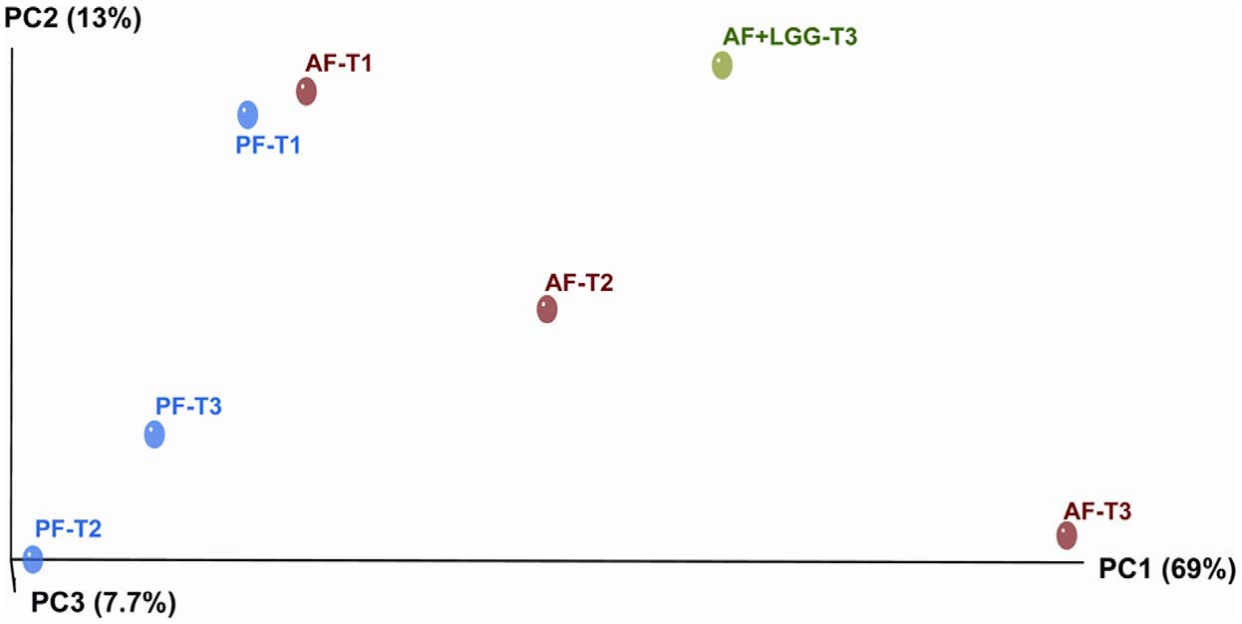
Weighted PCoA of bacterial genus by time and exposure group using UniFrac distance matrix. This dimension reduction analysis demonstrates the clustering of samples based on their location and similar microbial profiles. The first component explains 69% and the second component explains 13% of the variance in the samples. AF T3 group is the most divergent of the exposure groups while AF T2 and AF+LGG T3 are separated from the other baseline samples as well. AF T1, PF T1 are most similar as are PF T2 and T3.

The principal coordinate analysis (PCoA) gives a measure of bacterial genus community relatedness so that the samples with similar bacterial communities are localized in similar positions in the diagram. We observed a substantial separation in bacterial diversity at the genus level between the groups over time (Fig. 3). The baseline PF control group and the baseline AF group clustered in one coordinate space. At the end of 8 weeks, the AF group was the most divergent group in comparison to all other groups, suggesting that alcohol exposure over time leads to distinctly different composition of bacterial communities with respect to their genera and relative abundance. Further, the AF+LGG supplementation group clustered distinctly from the other groupsand was closer to the coordinate space of the T2 AF sample than the T3 AF sample. These data suggest that LGG supplementation decreased the shift in bacterial diversity occurring in response to alcohol feeding.

Further analysis of the specific bacterial genera that changed, showed that the bacterial genera that were most significantly overgrown in the alcohol exposed mice at the study endpoint compared to the PF control mice were *Corynebacterium* (group mean in AF = 0.164, std.err 0.005: group mean in PF = 0.001, std.err 0.0003: *p* value <0.001), *Alcaligenes* (group mean in AF = 0.181, std.err 0.005: group mean in PF = 0.001, std.err 0.0003: *p* value <0.001 ), *Listeria* (group mean in AF = 0.04, std.err 0.002: group mean in PF = 0.001, std.err 0.0004: *p* value <0.001), *Acetivibrio* (group mean in AF = 0.043, std.err 0.002: group mean in PF = 0.011, std.err 0.0001: *p* value <0.001), and *Allobaculum* (group mean in AF = 0.082, std.err 0.003: group mean in PF = 0.025, std.err 0.002: *p* value <0.001 ) (Table 2). The bacterial genera that were significantly displaced in the alcohol exposed mice at the study end point compared to the PF control mice were unclassified Bacteroides (group mean in AF = 0.004, std.err 0.0008: group mean in PF = 0.049, std.err 0.003: *p* value <0.001 ), *Tannerella* (group mean in AF = 0.021, std.err 0.001: group mean in PF = 0.089, std.err 0.004: *p* value <0.001), *Hallella* (group mean in AF = 0.009, std.err 0.001: group mean in PF = 0.063, std.err 0.003: *p* value <0.001 ), *unclassified Lachnospiraceae* (group mean in AF = 0.011, std.err 0.001: group mean in PF = 0.089, std.err 0.004: *p* value <0.001 ), and *Ruminococcaceae Incertae Sedis* (*undefined Ruminococcaceae*) (group mean in AF = 0.071, std.err 0.003: group mean in PF = 0.144, std.err 0.003: *p* value <0.001).

**Table 2:** Gains and loss of genera comparing AF and PF exposure groups at study endpoint (end of 8 weeks).

Genus	Phylum	Gram +/−	PF mean	PF Std.Err	AF mean	AF Std.Err	P val	Q val	up/down
*Bacteroides*	Bacteroidetes	n	0.049	0.003	0.004	0.0008	<0.001	<0.05	down
*Parabacteroides*	Bacteroidetes	n	0.040	0.002	0.021	0.001	<0.05	<0.05	down
*Tannerella*	Bacteroidetes	n	0.089	0.004	0.007	0.001	<0.001	<0.05	down
*Hallella*	Bacteroidetes	n	0.063	0.003	0.009	0.001	<0.001	<0.05	down
*Lachnospiraceae Other*	Firmicutes	p	0.089	0.004	0.011	0.001	<0.001	<0.05	down
*Ruminococcaceae Incertae Sedis*	Firmicutes	p	0.144	0.005	0.071	0.003	<0.001	<0.05	down
*Ruminococcaceae Other*	Firmicutes	p	0.045	0.003	0.011	0.001	<0.001	<0.05	down
***Corynebacterium***	**Actinobacteria**	**p**	**0.001**	**0.0003**	**0.164**	**0.005**	**<0.001**	**<0.05**	Up*
*Aerococcus*	Firmicutes	p	0.001	0.0004	0.106	0.004	<0.001	<0.05	up
*Listeria*	Firmicutes	p	0.001	0.0004	0.040	0.002	<0.001	<0.05	up
*Acetivibrio*	Firmicutes	n	0.011	0.001	0.043	0.002	<0.001	<0.05	up
*Clostridiales Other*	Firmicutes	p	0.048	0.003	0.092	0.004	<0.001	<0.05	up
*Allobaculum*	Firmicutes	p	0.025	0.002	0.082	0.003	<0.001	<0.05	up
*Lactobacillus*	Firmicutes	p	0.005	0.0009	0.009	0.001	<0.001	<0.05	up
***Alcaligenes***	**Proteobacteria**	**n**	**0.001**	**0.0003**	**0.181**	**0.005**	**<0.001**	**<0.05**	Up*

P value derived from t-test and q value derived from FDR.

**Figure 4:**
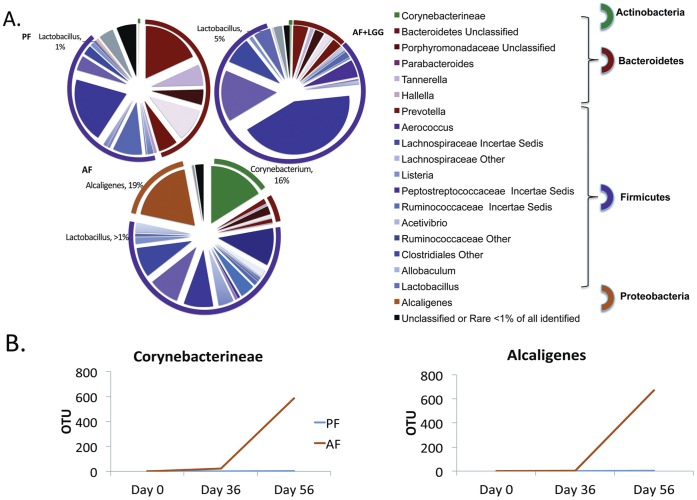
The relative distribution of the bacterial phyla and genera in response to ethanol feeding and LGG supplementation. (A) The microbiome of the PF (right), AF+LGG (left) and AF (middle lower) groups at T3 are shown in the pie charts and color coordinated by genus and phylum. The different shades of color represent the different genera and the common color spectrum (reds, purples, green and orange) represents the phyla. The outer ring around the pie charts also depicts the different phyla. The microbiomes of mice exposed to AF are characterized by greater abundance of *Alcaligenes* and *Corynebacterium* and loss of *Tannerella*. The AF+LGG group shows a much greater abundance of *Lactobacillus* and nonspecific *Ruminococcaceae Incertae Sedis* compared to the other exposure groups. (B) The growth rate over time of *Alcaligenes* and *Corynebacterium* in the PF and AF groups shows the expansion of the genera after exposure to alcohol.

The bacterial genera that showed the biggest expansion in response to chronic alcohol feeding was, *Corynebacterium* and *Alcaligenes*, which was not observed in the PF or AF+LGG groups. *Corynebacterium* and *Alcaligenes* also showed consistent growth in abundance over time (Fig. 4,) in response to alcohol exposure. The relative abundance of *Lactobacillus* was much greater in the AF+LGG (5%) group to the amount seen in the AF (<1%) group, and to the baseline (T1) PF diet group (1%). Quantitative PCR was performed with total bacterial 16S rRNA primers [21], [22] and Lactobacillus rhamnosus GG specific primers [23], [24] to confirm that the relative increase in Lactobacillus is specifically due to increased LGG (data not shown). The data show that there is a clear shift in the bacterial community in the alcohol exposed group, where there is a significant gain in the gram negative *Alcaligenes* and gram positive *Corynebacterium*, and suggests that LGG supplementation suppress the overgrowth of *Alicaligene* and *Corynebacterium*.

### Alcohol Feeding Increases Fecal pH

**Figure 5:**
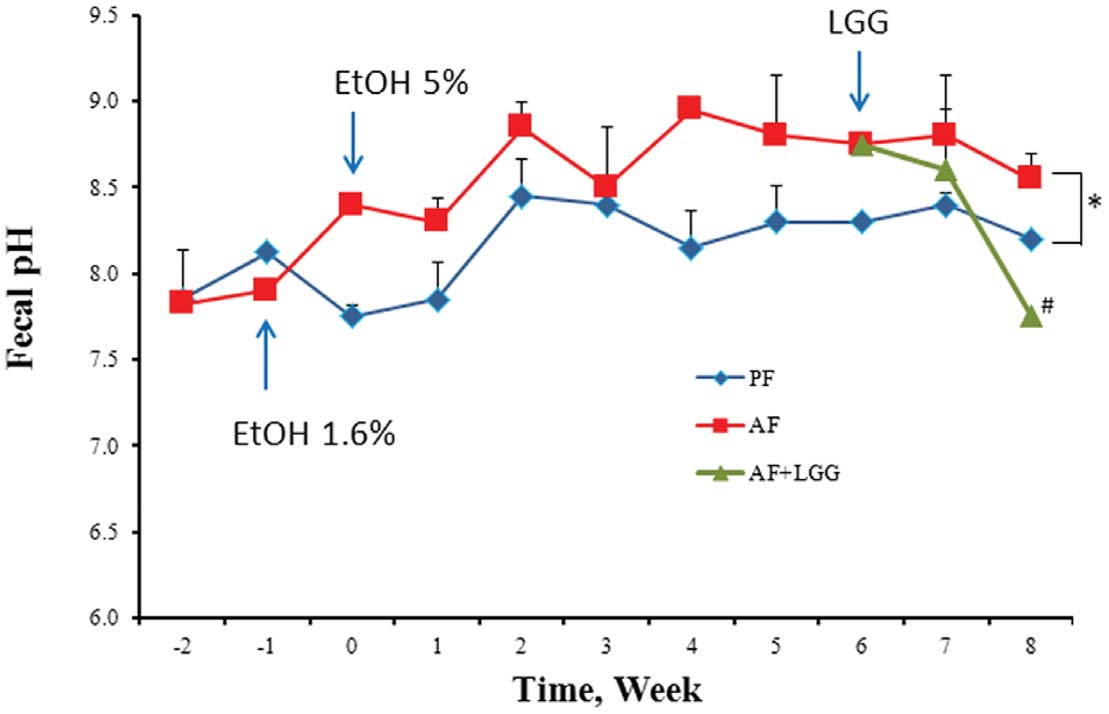
Fecal pH changes in response to alcohol feeding and LGG supplementation. Animals were fed Lieber-DeCarli liquid diet with alcohol (AF) or without (PF). LGG supplementation (AF+LGG) was started at the end of week 6 and continued till the end of the experiment (8 weeks). Fecal samples were collected weekly. Data was obtained from n = 6–8 for the PF and AF groups and n = 4 for the AF+LGG group. Fecal pH significantly increased in response to alcohol feeding over the study period of 8 weeks (*p<0.0001, AF versus PF). Comparison of fecal samples obtained from LGG supplemented animals (AF+LGG) with alcohol fed animals (AF) obtained at the end of the study period (samples at the end of week 8) showed a significant decrease in fecal pH (# p = 0.001, AF versus AF+LGG at week 8).

Previous studies have shown that several pathological conditions increase colon pH. Changes in the luminal environmental pH potentially alter the competitive advantage for the various bacterial communities. The effect of alcohol on the lower colon pH was examined by measuring pH of the fecal samples. Commensurate with the expansion of the *Alcaligenes* genus that represents the bacteria requiring higher/alkaline pH, alcohol exposure indeed increased fecal pH over time. Importantly, LGG supplementation which prevented the expansion of the *Alcaligenes* was also able to significantly reduce the fecal pH (Fig. 5). Our data indicates there is a significant difference in the luminal environmental pH when the LGG probiotic is added.

### LGG Supplementation Corrects the Decrease in Tight Junction Protein Expression and Attenuates Endotoxemia and Hepatic Injury in Alcohol Fed Animals

**Figure 6:**
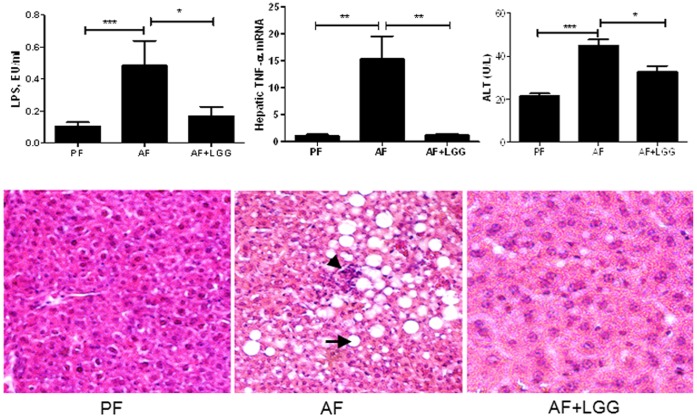
LGG probiotic supplementation ameliorates alcohol-induced blood endotoxemia, hepatic inflammation, steatosis and injury. Data was obtained from n = 6–8 for the PF and AF groups and n = 4 for the AF+LGG group. (A) Plasma endotoxin levels assessed by LPS (lipopolysaccharides) measurement. Two weeks of LGG supplementation attenuated alcohol-mediated increase in plasma LPS levels. (B) Hepatic marker of inflammation, TNF-α m-RNA, was significantly up-regulated in response to alcohol feeding. LGG supplementation was able to bring it down to the level of control pair-fed animals. (C) Liver injury was evaluated by plasma ALT activity. Alcohol feeding resulted in significant elevation of plasma ALT levels compared to control pair-fed animals, which was decreased by LGG treatment. (D) Hematoxylineosin staining demonstrated severe micro- and macrovesicular fat accumulation in the livers of alcohol fed mice compared to control pair-fed animals. Two weeks of LGG treatment markedly attenuated alcohol-induced hepatic steatosis. Arrow indicates the fat droplets and arrow head denotes neutrophil infiltration foci (x200 final magnification). *p<0.05; **p<0.01; ***p<0.001.

Tight junction (TJ) proteins play a critical role in maintaining the gut barrier integrity and can be affected by ethanol exposure leading to gut barrier dysfunction and an increase in intestinal permeability. Our recent work has shown that chronic ethanol exposure disrupts TJ proteins [19]; hence, in the present study mRNA expression of key markers of TJ integrity, as well as several TJ protein adaptors were evaluated. Significant down-regulation in the mRNA expression of ZO-1 and claudin-1, and adaptors symplekin, p130 and fordin was observed in response to chronic ethanol feeding which was corrected by LGG supplementation (Table 3). Further, commensurate with the expansion of the *Proteobacteria* phylum (containing Gram negative bacteria) and impaired gut barrier function, chronic ethanol feeding increased plasma endotoxin/lipopolysaccharide (LPS) levels (Fig. 6A). Since endotoxemia positively correlates with hepatic inflammation and injury, these parameters were assessed. Along with endotoxemia, ethanol feeding also increased hepatic inflammation as shown by increased hepatic TNF-α expression (Fig. 6B), and caused injury as seen by significantly increased ALT levels (Fig. 6C). Moreover, hepatic steatosis which is a precursor to both inflammation and injury was also significantly increased (Fig. 6D). Importantly, ethanol mediated endotoxemia, along with hepatic inflammation, injury and steatosis were markedly attenuated by treatment with LGG.

**Table 3:** Effect of LGG supplementation on mRNA levels of intestinal tight junction proteins and protein adaptors.

TJs and adaptors	PF	AF	AF+LGG
ZO-1	1.477±0.396 (5)	0.659±0.239* (7)	2.186±0.737** (4)
Claudin-1	0.914±0.214 (4)	0.419±0.0769* (6)	0.941±0.204** (4)
Occludin	1.367±0.342 (5)	0.681±0.341 (6)	1,929±0.767 (4)
Symplekin	1.240±0.276 (6)	0.455±0.121* (6)	1.217±0.274** (4)
p130	1.033±0.147 (5)	0.317±0.0591* (5)	0.789±0.175** (4)
Fordin	1.577±0.447 (6)	0.589±0.220 (7)	1.867±0.710 (4)

Results are means ± SEM.

* significantly different vs. PF;

** significantly different vs. AF. (Note: the number in the parentheses is the number of observations for each data point).

## Discussion

Our results demonstrate that in a mouse model of alcoholic liver disease, chronic alcohol feeding leads to a significant change in the intestinal bacterial community structure over time. Chronic alcohol feeding causes a select expansion of microbial membership, intestinal permeability and bacterial translocation/endotoxemia. Our results also indicate that the ethanol feeding-mediated loss of the native bacteria is correlated with a change in luminal pH that may drive the alterations in the microbial membership. Furthermore, we have shown that this change can be mitigated with LGG probiotic treatment.

Modulation of the intestinal microbiota by using probiotic treatment is a developing strategy to reduce bacterial translocation and circulating endotoxin levels. Treatment with probiotics has been successfully shown to reduce the Gram-negative bacterial population in multiple studies modeling alcohol induced liver disease in rats [5], [7], [10]. We have reported similar findings in humans as well [25]. In a study on a cohort of Russian men admitted to a psychiatric hospital with a diagnosis of alcoholic psychosis, short-term oral supplementation with probiotics was associated with restoration of the bowel microbiome and greater improvement in alcohol-induced liver injury than standard therapy alone. Our recent study examining the mechanisms of how *Lactobacillus* attenuates ALD showed that LGG supplementation prevents alcohol-induced decrease in the expression of intestinal trefoil factor and its transcriptional regulator hypoxia-inducible factor-2α (HIF-2α) and resultant epithelial barrier dysfunction [19]. Hence, in the present study, the remodeling of the microbial community structure in response to LGG probiotic treatment, maybe a contributing factor causing a decrease in intestinal permeability and consequently attenuating bacterial translocation and endotoxemia, hepatic TNF-α levels and steatosis and injury. These data strongly suggest that the ethanol mediated expansion of the *Proteobacteria* and *Actinobacteria* plays a pathogenic role in the development of ALD (intestinal permeability, endotoxemia, and hepatic inflammation and injury) and can be prevented by the LGG probiotic treatment. Overall, this study supports modulation of intestinal microbiota as a potential therapeutic intervention in the treatment of alcohol-induced endotoxemia and liver disease.

The presence and significant role of systemic endotoxemia in the development of hepatic inflammation and injury is well described in alcoholic patients and animal models of alcoholic liver disease [1]–[3], [5], [6], [10]. The ALD associated endotoxemia is due to an enhancement in the intestinal permeability which facilitates the escape of bacterial endotoxin and resultant increase into the portal circulation [4], [8], [26]. Along with alcohol, higher than normal amounts of endotoxin can prime and activate both hepatic macrophages (Kupffer cells) and extrahepatic macrophages to overproduce inflammatory cytokines such as TNF-α, IL-6, and IL-8 [27], [28]. Systemic endotoxemia *per se* and endotoxin-induced inflammatory cytokines further increase intestinal permeability through modulation of intestinal tight junctions and the vicious cycle of endotoxemia is maintained [29]. In context of this study, the evaluation of the composition of the microbial membership showed that ethanol treatment caused a decline in the abundance of both *Bacteriodetes* and *Firmicutes*, with *Bacteriodetes* showing a pronounced reduction compared to *Firmicutes*. Further, the decrease in *Bacteroidetes* and *Firmicutes* was accompanied by a proportional increase in *Actinobacteria* and *Proteobacteria*. The expansion of these bacteria is significant because the phylum *Proteobacteria* includes Gram negative bacteria which includes a wide variety of pathogenic species, such as *Escherichia*, *Salmonella*, *Vibrio*, and *Helicobacter* [11]. These results strongly indicate that the increase in plasma endotoxin levels and hepatic inflammation is a consequence of the expansion of the gram-negative bacteria from the *Proteobacteria* phylum, which occur in response to chronic ethanol consumption and can be prevented by the LGG probiotic treatment.

The stability of the normal intestinal microbiome is influenced by several factors in the luminal environment including gastric acidity, gut motility, bile salts, immunological defense factors, colonic pH and competition between micro-organisms for nutrients and intestinal binding sites [30]. Altered luminal environment may lead to modifications in the microbial membership by supporting the growth of specific genera. Accordingly, a remarkable increase of *Alcaligenes,* an alkaline tolerant genus from the *Proteobacteria* phylum, occurred in response to chronic alcohol feeding, suggesting alterations in the luminal environment are due to increased pH. Indeed, the increased abundance of *Alcaligenes* strongly correlated with increased fecal pH. These data suggest that increased luminal pH may be a significant contributing factor in the ethanol-induced alterations in the intestinal microbiota. Increased luminal pH, leading to pathogenic alterations, is implicated in diverse disease states including infantile diarrhea and liver cirrhosis [31], [32]. With regards to the protective effects of probiotic treatment using LGG several mechanisms have been proposed. In particular, the mechanisms by which LGG may exert its protective effects in different pathogenic conditions include inhibition of the mucosal adhesion of pathogens, improvement of the intestinal epithelial barrier function, alteration of the host innate and adaptive immune activity, competition for nutrients and reduction of the luminal pH through short chain fatty acid (SCFA) production [33], [34]. In relevance to the current findings, several studies have shown a correlation between lowering of fecal pH and inhibition of the growth of harmful and pathogenic organisms [31]. Hence, the inhibition of the ethanol-induced expansion of the *Proteobacteria* and *Actinobacteria* phyla by LGG probiotic treatment in our study has likely resulted from the formation of SCFA and lowering of luminal pH as reflected by a decrease and normalization of the fecal pH. Additionally, we also observed an increase in the *Corynebacterium* genus, a member of the *Actinobacteria* phylum that dominated the community structure over time. Multiple studies have described opportunistic infections of *Corynebacterium* members in subjects that have a history of alcohol derived liver disease [35], [36], suggesting that this bacteria may commonly overgrow in a system chronically exposed to alcohol. The relevance of the increase of *Corynebacterium* in the context of this study is currently unknown. Further research is needed to identify how the local environment provides a metabolic advantage to one community member compared to another. However, the present findings are suggestive of a mechanism whereby the chronic alcohol exposure increases the pH in the gut allowing the expansion of certain specific bacteria like *Alcaligenes* or *Corynebacterium* or similar bacteria that can have a competitive growth advantage. Since treatment with LGG decreased the ethanol induced fecal pH, it is conceivable that the ability of lactobacilli to produce SCFAs and lowering luminal pH prevented the pathogenic expansion of *Proteobacteria* and *Actinobacteria* phyla.

Our observation of a decrease in *Bacteroidetes* is consistent with other inflammatory disease models. Particularly, analysis of the bacterial genomic DNA extracted from colonic biopsy specimens showed a significantly lower abundance of *Bacteroidetes* and higher abundance of *Proteobacteria* in individuals with colorectal adenomas compared to healthy controls, which is thought to contribute to the etiology of colorectal cancer [37]. Further, a survey of the distal gut microbiota of adult C57BL/6J genetically obese (ob/ob) mice showed that the relative abundance of the *Bacteroidetes* was lower by 50%, whereas the *Firmicutes* were higher by a corresponding degree [38], [39]. Similarly, in a study describing the changes in murine model’s microbiome in liver disease, researchers found a significant increase in *Firmicutes* and *Actinobacteria* in mice that were treated with carbon tetrachloride that was designed to induce bridging fibrosis liver injury [40]. In this study, the ethanol feeding mediated decrease in *Bacteroidetes* was not compensated by an increase in *Firmicutes* but rather an increase in *Proteobacteria* and *Actinobacteria*.

To date, there are few papers published on the effect of alcohol on the microbiome. In the present study, there was a significant decrease in *Bacteroidetes*. Our results are consistent with those results observed in humans. Mutlu et.al. observed the same decrease in *Bacteroidetes* and increase in *Proteobacteria* in alcoholic humans with dysbiosis [41]. However, an expansion in *Bacteroidetes* was observed by Yan et.al in ethanol treated mice [9]. It should be noted that in the study by Yan et al., ethanol treatment was carried out via intragastric feeding for 3 weeks compared to *ad libidum* Leiber DeCarli ethanol diet for 8 weeks in the current study. The 3 week bacterial population in the Yan et al., study may represent the microbial community structure in flux but not the extent or ultimate direction of the flux. Other research also suggests the effects of alcohol on the changes in dysbiosis takes a longer time period than 3 weeks [7].

In summary, our results show an important change in the bacterial composition over time in alcohol fed mice, which corresponds to increases in gut permeability, plasma LPS levels and hepatic inflammatory markers. We have also shown that the altered bacterial communities of the gut may be a significant therapeutic target for the prevention/treatment of intestinal barrier dysfunction and consequent alcoholic liver disease.

## References

[1] Adachi Y, Moore LE, Bradford BU, Gao W, Thurman RG (1995) Antibiotics prevent liver injury in rats following long-term exposure to ethanol. Gastroenterology 108: 218–224. 7806045 10.1016/0016-5085(95)90027-6

[2] Bode C, Kugler V, Bode JC (1987) Endotoxemia in patients with alcoholic and non-alcoholic cirrhosis and in subjects with no evidence of chronic liver disease following acute alcohol excess. Journal of hepatology 4: 8–14. 3571935 10.1016/s0168-8278(87)80003-x

[3] Fukui H, Brauner B, Bode JC, Bode C (1991) Plasma endotoxin concentrations in patients with alcoholic and non-alcoholic liver disease: reevaluation with an improved chromogenic assay. Journal of hepatology 12: 162–169. 2050995 10.1016/0168-8278(91)90933-3

[4] Keshavarzian A, Choudhary S, Holmes EW, Yong S, Banan A, et al (2001) Preventing gut leakiness by oats supplementation ameliorates alcohol-induced liver damage in rats. The Journal of pharmacology and experimental therapeutics 299: 442–448. 11602653

[5] Nanji AA, Khettry U, Sadrzadeh SM (1994) Lactobacillus feeding reduces endotoxemia and severity of experimental alcoholic liver (disease). Proceedings of the Society for Experimental Biology and Medicine Society for Experimental Biology and Medicine 205: 243–247. 10.3181/00379727-205-43703 8171045

[6] Parlesak A, Schafer C, Schutz T, Bode JC, Bode C (2000) Increased intestinal permeability to macromolecules and endotoxemia in patients with chronic alcohol abuse in different stages of alcohol-induced liver disease. Journal of hepatology 32: 742–747. 10845660 10.1016/s0168-8278(00)80242-1

[7] Mutlu E, Keshavarzian A, Engen P, Forsyth CB, Sikaroodi M, et al (2009) Intestinal dysbiosis: a possible mechanism of alcohol-induced endotoxemia and alcoholic steatohepatitis in rats. Alcoholism, clinical and experimental research 33: 1836–1846. 10.1111/j.1530-0277.2009.01022.x PMC3684271 19645728

[8] Rao RK, Seth A, Sheth P (2004) Recent Advances in Alcoholic Liver Disease I. Role of intestinal permeability and endotoxemia in alcoholic liver disease. American journal of physiology Gastrointestinal and liver physiology 286: G881–884. 15132946 10.1152/ajpgi.00006.2004

[9] Yan AW, Fouts DE, Brandl J, Starkel P, Torralba M, et al (2011) Enteric dysbiosis associated with a mouse model of alcoholic liver disease. Hepatology 53: 96–105. 21254165 10.1002/hep.24018 PMC3059122

[10] Forsyth CB, Farhadi A, Jakate SM, Tang Y, Shaikh M, et al (2009) Lactobacillus GG treatment ameliorates alcohol-induced intestinal oxidative stress, gut leakiness, and liver injury in a rat model of alcoholic steatohepatitis. Alcohol 43: 163–172. 19251117 10.1016/j.alcohol.2008.12.009 PMC2675276

[11] Madigan MT (2006) Chapter 12 Prokaryotic Diversity: The Bacteria Chapter. In: Martinko JM, editor. Brock Biology of Microorganisms. 11 ed. Upper Saddle River, NJ: Pearson Prentice Hall.

[12] HMP-Consortium (Accepted-2012) Evaluation of 16S rDNA-based community profiling for human microbiome research. PLoS One. 10.1371/journal.pone.0039315 PMC3374619 22720093

[13] Haas BJ, Gevers D, Earl AM, Feldgarden M, Ward DV, et al (2011) Chimeric 16S rRNA sequence formation and detection in Sanger and 454-pyrosequenced PCR amplicons. Genome Res 21: 494–504. 21212162 10.1101/gr.112730.110 PMC3044863

[14] Maidak BL, Olsen GJ, Larsen N, Overbeek R, McCaughey MJ, et al (1997) The RDP (Ribosomal Database Project). Nucleic acids research 25: 109–111. 9016515 10.1093/nar/25.1.109 PMC146422

[15] Schloss PD, Westcott SL, Ryabin T, Hall JR, Hartmann M, et al (2009) Introducing mothur: open-source, platform-independent, community-supported software for describing and comparing microbial communities. Appl Environ Microbiol 75: 7537–7541. 19801464 10.1128/AEM.01541-09 PMC2786419

[16] Kuczynski J, Stombaugh J, Walters WA, Gonzalez A, Caporaso JG, et al.. (2011) Using QIIME to analyze 16S rRNA gene sequences from microbial communities. Current protocols in bioinformatics/editoral board, Andreas D Baxevanis [et al] Chapter 10: Unit 10 17. 10.1002/0471250953.bi1007s36 PMC3249058 22161565

[17] Lozupone C, Hamady M, Knight R (2006) UniFrac–an online tool for comparing microbial community diversity in a phylogenetic context. BMC bioinformatics 7: 371. 16893466 10.1186/1471-2105-7-371 PMC1564154

[18] White JR, Nagarajan N, Pop M (2009) Statistical methods for detecting differentially abundant features in clinical metagenomic samples. PLoS computational biology 5: e1000352. 19360128 10.1371/journal.pcbi.1000352 PMC2661018

[19] Wang Y, Kirpich I, Liu Y, Ma Z, Barve S, et al (2011) Lactobacillus rhamnosus GG treatment potentiates intestinal hypoxia-inducible factor, promotes intestinal integrity and ameliorates alcohol-induced liver injury. The American journal of pathology 179: 2866–2875. 22093263 10.1016/j.ajpath.2011.08.039 PMC3260853

[20] Abou-Donia MB, El-Masry EM, Abdel-Rahman AA, McLendon RE, Schiffman SS (2008) Splenda alters gut microflora and increases intestinal p-glycoprotein and cytochrome p-450 in male rats. Journal of toxicology and environmental health Part A 71: 1415–1429. 18800291 10.1080/15287390802328630

[21] Maeda H, Fujimoto C, Haruki Y, Maeda T, Kokeguchi S, et al (2003) Quantitative real-time PCR using TaqMan and SYBR Green for Actinobacillus actinomycetemcomitans, Porphyromonas gingivalis, Prevotella intermedia, tetQ gene and total bacteria. FEMS Immunol Med Microbiol 39: 81–86. 14557000 10.1016/S0928-8244(03)00224-4

[22] Horz HP, Vianna ME, Gomes BP, Conrads G (2005) Evaluation of universal probes and primer sets for assessing total bacterial load in clinical samples: general implications and practical use in endodontic antimicrobial therapy. J Clin Microbiol 43: 5332–5337. 16208011 10.1128/JCM.43.10.5332-5337.2005 PMC1248440

[23] Brandt K, Alatossava T (2003) Specific identification of certain probiotic Lactobacillus rhamnosus strains with PCR primers based on phage-related sequences. Int J Food Microbiol 84: 189–196. 12781941 10.1016/s0168-1605(02)00419-1

[24] Dommels YE, Kemperman RA, Zebregs YE, Draaisma RB, Jol A, et al (2009) Survival of Lactobacillus reuteri DSM 17938 and Lactobacillus rhamnosus GG in the human gastrointestinal tract with daily consumption of a low-fat probiotic spread. Appl Environ Microbiol 75: 6198–6204. 19684171 10.1128/AEM.01054-09 PMC2753077

[25] Kirpich IA, Solovieva NV, Leikhter SN, Shidakova NA, Lebedeva OV, et al (2008) Probiotics restore bowel flora and improve liver enzymes in human alcohol-induced liver injury: a pilot study. Alcohol 42: 675–682. 19038698 10.1016/j.alcohol.2008.08.006 PMC2630703

[26] Ferrier L, Berard F, Debrauwer L, Chabo C, Langella P, et al (2006) Impairment of the intestinal barrier by ethanol involves enteric microflora and mast cell activation in rodents. The American journal of pathology 168: 1148–1154. 16565490 10.2353/ajpath.2006.050617 PMC1606551

[27] Gobejishvili L, Barve S, Joshi-Barve S, McClain C (2008) Enhanced PDE4B expression augments LPS-inducible TNF expression in ethanol-primed monocytes: relevance to alcoholic liver disease. American journal of physiology Gastrointestinal and liver physiology 295: G718–724. 18687753 10.1152/ajpgi.90232.2008 PMC2575909

[28] McClain CJ, Barve S, Deaciuc I, Kugelmas M, Hill D (1999) Cytokines in alcoholic liver disease. Seminars in liver disease 19: 205–219. 10422201 10.1055/s-2007-1007110

[29] Ma TY, Iwamoto GK, Hoa NT, Akotia V, Pedram A, et al (2004) TNF-alpha-induced increase in intestinal epithelial tight junction permeability requires NF-kappa B activation. American journal of physiology Gastrointestinal and liver physiology 286: G367–376. 14766535 10.1152/ajpgi.00173.2003

[30] Fooks LJ, Gibson GR (2002) Probiotics as modulators of the gut flora. The British journal of nutrition 88 Suppl 1S39–49. 12215180 10.1079/BJN2002628

[31] Bezkorovainy A (2001) Probiotics: determinants of survival and growth in the gut. The American journal of clinical nutrition 73: 399S–405S. 11157348 10.1093/ajcn/73.2.399s

[32] Zhao HY, Wang HJ, Lu Z, Xu SZ (2004) Intestinal microflora in patients with liver cirrhosis. Chinese journal of digestive diseases 5: 64–67. 15612659 10.1111/j.1443-9573.2004.00157.x

[33] Saxelin M (2002) Lactobacillus GG and its health effects Finland: Valio Ltd,.

[34] Drisko JA, Giles CK, Bischoff BJ (2003) Probiotics in health maintenance and disease prevention. Altern Med Rev 8: 143–155. 12777160

[35] Cericco M, Iglicki F, Guillaumont MP, Schmitt JL, Dupas JL, et al (1996) [Corynebacterium xerosis endocarditis associated with alcoholic cirrhosis]. Gastroenterologie clinique et biologique 20: 514. 8761156

[36] Harnisch JP, Tronca E, Nolan CM, Turck M, Holmes KK (1989) Diphtheria among alcoholic urban adults. A decade of experience in Seattle. Annals of internal medicine 111: 71–82. 2472081 10.7326/0003-4819-111-1-71

[37] Shen XJ, Rawls JF, Randall T, Burcal L, Mpande CN, et al (2010) Molecular characterization of mucosal adherent bacteria and associations with colorectal adenomas. Gut microbes 1: 138–147. 20740058 10.4161/gmic.1.3.12360 PMC2927011

[38] Ley RE, Backhed F, Turnbaugh P, Lozupone CA, Knight RD, et al (2005) Obesity alters gut microbial ecology. Proc Natl Acad Sci U S A 102: 11070–11075. 16033867 10.1073/pnas.0504978102 PMC1176910

[39] Turnbaugh PJ, Ley RE, Mahowald MA, Magrini V, Mardis ER, et al (2006) An obesity-associated gut microbiome with increased capacity for energy harvest. Nature 444: 1027–1031. 17183312 10.1038/nature05414

[40] Fouts DE, Torralba M, Nelson KE, Brenner DA, Schnabl B (2012) Bacterial translocation and changes in the intestinal microbiome in mouse models of liver disease. Journal of hepatology. 10.1016/j.jhep.2012.01.019 PMC3357486 22326468

[41] Mutlu EA, Gillevet PM, Rangwala H, Sikaroodi M, Naqvi A, et al (2012) Colonic microbiome is altered in alcoholism. American journal of physiology Gastrointestinal and liver physiology 302: G966–978. 22241860 10.1152/ajpgi.00380.2011 PMC3362077

